# Evolution of ASC Immunophenotypical Subsets During Expansion In Vitro

**DOI:** 10.3390/ijms21041408

**Published:** 2020-02-19

**Authors:** Qiuyue Peng, Hiva Alipour, Simone Porsborg, Trine Fink, Vladimir Zachar

**Affiliations:** Department of Health Science and Technology, Regenerative Medicine Group, Aalborg University, Fredrik Bajers Vej 3B, 9220 Aalborg, Denmark; qp@hst.aau.dk (Q.P.); hiva@hst.aau.dk (H.A.); sriis@hst.aau.dk (S.P.); trinef@hst.aau.dk (T.F.)

**Keywords:** adipose-derived stem cells, immunophenotype, co-expression, subpopulations, regenerative medicine

## Abstract

Adipose-derived stromal/stem cells (ASCs) are currently being considered for clinical use for a number of indications. In order to develop standardized clinical protocols, it is paramount to have a full characterization of the stem cell preparations. The surface marker expression of ASCs has previously been characterized in multiple studies. However, most of these studies have provided a cross-sectional description of ASCs in either earlier or later passages. In this study, we evaluate the dynamic changes of 15 different surface molecules during culture. Using multichromatic flow cytometry, ASCs from three different donors each in passages 1, 2, 4, 6, and 8 were analyzed for their co-expression of markers associated with mesenchymal stem cells, wound healing, immune regulation, ASC markers, and differentiation capacity, respectively. We confirmed that at an early stage, ASC displayed a high heterogeneity with a plethora of subpopulations, which by culturing became more homogeneous. After a few passages, virtually all ASCs expressed CD29, CD166 and CD201, in addition to canonical markers CD73, CD90, and CD105. However, even at passage 8, there were several predominant lineages that differed with respect to the expression of CD34, CD200 and CD271. Although the significance of remaining subpopulations still needs to be elucidated, our results underscore the necessity to fully characterize ASCs prior to clinical use.

## 1. Introduction

Adipose-derived stromal/stem cells (ASCs) are a favorable type of mesenchymal stem cell (MSC) due to easy accessibility, high cell yield, and robust proliferative capacity [1,2]. ASCs have been recognized to be highly biologically active, with regenerative roles in tissue formation, homeostasis, and immune regulation [3,4,5]. These unique properties render ASCs attractive in scenarios where the traditional approaches fall short of the desired therapeutic outcome, such as for chronic wounds, diabetic ulcers, osteoarthritis, ischemic heart disease, or type 1 diabetes, just to mention a few important areas [6,7,8,9,10]. Many ASC-based regimens have already progressed into clinical testing, and in the United States alone, more than 20 phase I and II clinical trials are currently being conducted (clinicaltrials.gov). All of the protocols are based on the use of crude ASC cultures, which are heterogeneous mixtures containing various stem and progenitor cells that differ functionally and immunophenotypically. Additionally, the properties and ratios of these subpopulations have been shown to change along with in vitro expansion [11]. It is unlikely that all lineages within the cell preparation participate to the same extent in the therapeutic effect [12]. Provided that the discrete subpopulations can be linked with particular functionalities, it would be possible in the prospective cell-based protocols to tailor the desired properties to the clinical application, which would undoubtedly benefit the clinical outcome.

The analysis of surface epitope expression is a powerful tool to identify and isolate stem cells. Initially, in addition to plastic adherence and capacity for tri-lineage differentiation, a very specific triad of cluster of differentiation (CD) molecules CD73, CD90, and CD105 was claimed to represent the quintessential ASC hallmark [13]. However, the ensuing research lacks consensus in regard to the full stem cell marker profile of ASCs [1,14,15,16]. Additionally, many other molecules have been reported in association with ASCs, but their expression still remains controversial and is believed to be affected by donor-to-donor variability, differing protocols of isolation and expansion, and diverse experimental techniques [17,18,19,20,21,22]. In addition to investigations aiming at the descriptive characterization of the surface marker expression, a great effort was dedicated to elucidating the relationship between the phenotypical features and the functional properties. Indeed, some phenotypes displaying specific patterns of positivity and negativity for a limited number of selected markers have been found to possess an enhanced functionality, such as increased differentiation potential, pro-angiogenic effect, and higher proliferative rate or modulation of the immune response [15,23,24,25,26,27,28,29].

In our previous work, while looking at six CD markers, we demonstrated that only 8% of all possible combinations were present [12]. Thus, it is plausible that the heterogeneity of ASC cultures is limited with only a restricted number of variants, depending on the permissive combinations of the CD markers. To shed more light on the possible combinatorial repertoires, we, in the current study, based on a thorough literature search, highly expanded the number of membrane molecules to 15 markers and examined their triple co-expression according to functional grouping into 5 categories, including MSC markers, wound healing, immune regulation, ASC markers, and differentiation capacity [24,25,27,28,29,30,31,32,33,34,35,36,37,38,39,40,41,42,43,44,45]. This was done in the search for better insight into the dynamic changes of the immunophenotypical subsets and to elucidate the significance of the choice of donor and the effect of culture expansion. We found that at an early stage, ASCs displayed a high heterogeneity with a plethora of subpopulations, which by culturing became homogenized due to three ways of re-distribution: exchange, converge, or diverge. This was independent of the choice of donor; however, donor-dependent differences in the dynamic of some ASC subsets were discovered.

## 2. Results

### 2.1. Temporal Changes of ASC Surface Markers during In Vitro Expansion

**Figure 1:**
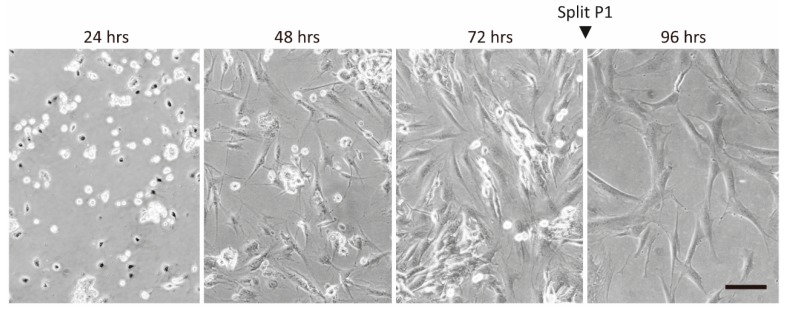
Development of adipose-derived stromal/stem cell (ASC) morphology after isolation (P0), and establishment of passage 1 (P1). Original magnification 200×. Scale bar indicates 100 µm.

Stromal vascular fractions (SVF) from the three donors were seeded and expanded at standard cell culture conditions for 8 passages (P) with an average of 1.76 population doublings per passage. The cultures underwent the most dramatic morphological changes during the first expansion, where the cells evolved from pleiomorphic appearance to more uniform, fibroblast-resembling types in the early P1 (Figure 1).

To study the immunophenotypical changes of ASCs during the expansion period, 15 markers, including CD73, CD90, CD105, CD248, CD200, CD201, CD36, CD274, CD29, CD166, CD34, CD146, CD31, CD27, and the Stro-1, were selected based on their relevance for ASCs and their proposed functionality. For the analysis, the markers were sorted by function into five panels: MSC markers, wound healing, immune regulation, ASC markers, and differentiation capacity (Table 1). Each panel thus consisted of three markers, which were then assayed simultaneously using multi-color flow cytometry. This setup enabled the follow-up of expressional kinetics of individual markers as well as the evolution of more complex co-expressional patterns.

**Figure 2:**
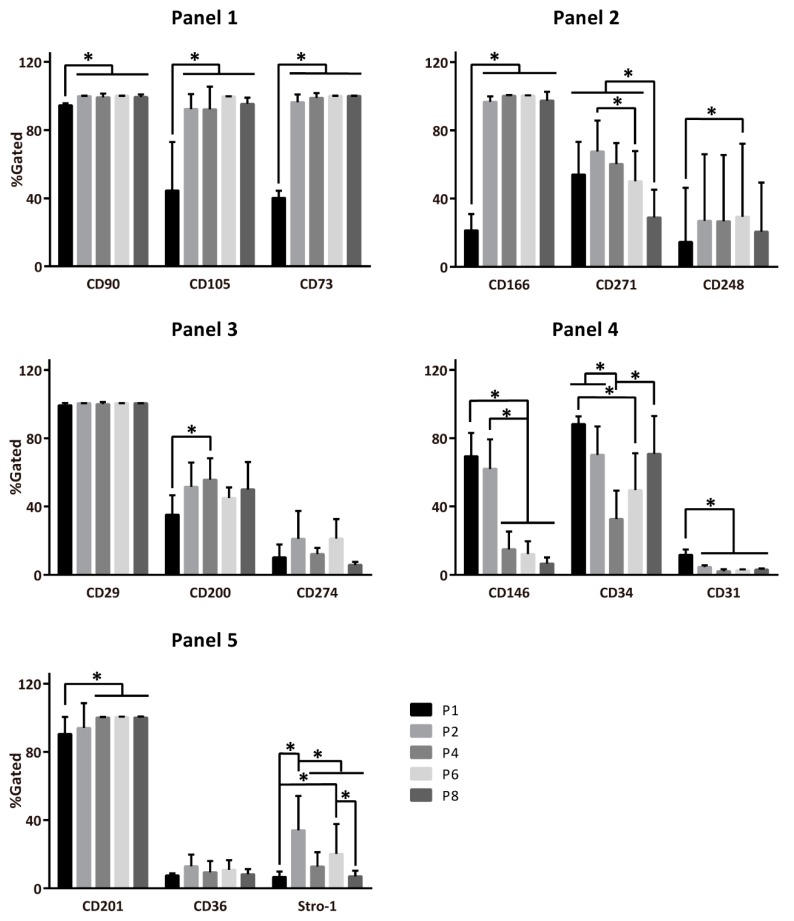
Development of single-marker expression across population expansion. Panels: 1, MSC markers; 2, wound healing markers; 3, immune regulation markers; 4, ASC markers, 5, differentiation capacity markers. ASC; adipose-derived stromal/stem cells, CD; cluster of differentiation, Passage 1–8 (P1–8). The data are presented as means + standard deviation, *n* = 7–8, * indicates a statistically significant change *p* < 0.05.

When looking at the expression of single markers, only the integrin β-1 (CD29; Panel 3) was consistently expressed, irrespective of the culture period (Figure 2). Not surprisingly, the MSC prototypical markers from Panel 1 stabilized with culture time, but the CD166 and CD201 from Panels 2 and 5, respectively, also followed this pattern. The ASC markers in Panel 4 generally diminished during the culture, whereas the markers associated with wound healing (Panel 2), immune regulation (Panel 3), and differentiation (Panel 5) remained expressed at variable levels throughout the culture period.

**Figure 3:**
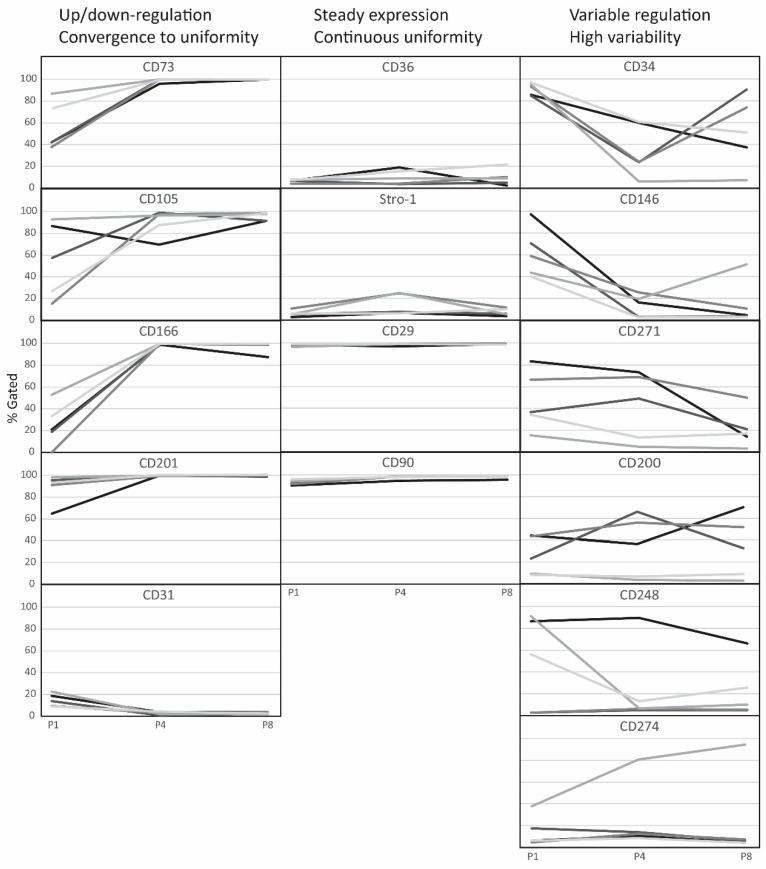
Kinetic profiles of single-marker expression across population expansion. Traces from 5 donors are shown at passages P1, P4, and P8. CD; cluster of differentiation, P; passage.

Based on kinetic profiles, it was possible to classify the markers into three categories, where the spread of values converged during the culture onto a highly predictable value, remained constantly low irrespective of high or low expression levels, or was high due to individually markedly discordant expression patterns, as shown in Figure 3.

### 2.2. Evolution of Co-Expression Patterns

**Figure 4:**
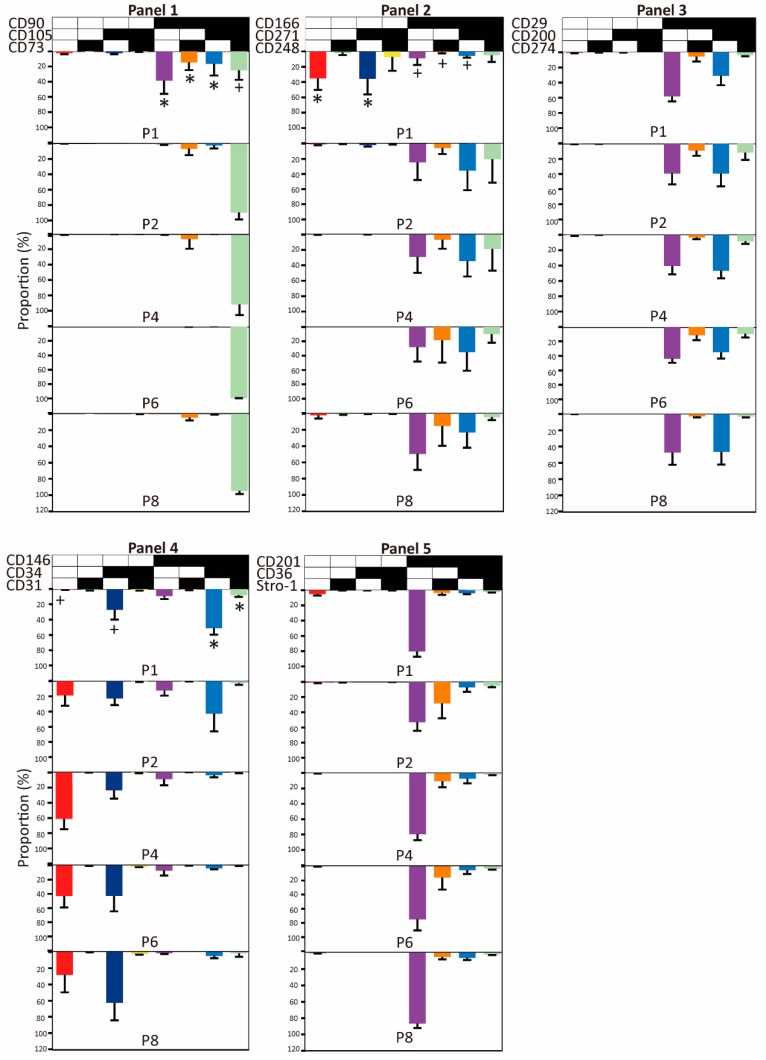
The prevalence of complex immunophenotypes within the functional panels and their temporal changes over eight passages. Panels: 1, mesenchymal stem cell (MSC) markers; 2, wound healing markers; 3, immune regulation markers; 4, Adipose-derived stromal/stem cell (ASC) markers, 5, differentiation capacity markers. A black field indicates that the marker was expressed; A white field indicates that the marker was not expressed. The data are presented as means + standard deviation, *n* = 7–8. *: population with the given combination of markers significantly decreases during culture. +: population with the given combination of markers significantly increases during culture. CD; cluster of differentiation.

The prevalence of complex immunophenotypes within the functional panels and their temporal changes over eight passages are presented in Figure 4. Overall, only a limited number of variants out of all possible marker combinations was identified, and two distinct evolutionary patterns were discernible. In the majority of cases (Panels 1, 2, and 4), the spectrum of examined triple combinations was rather broad early in the culture (P1) but became rapidly reduced at the following passage or P4 at the latest. Interestingly, these changes are also coincident with the increase in the uniformity of cell cultures, as described in the previous section. The selected combinations then continued being stably expressed for the remainder of the culture. In the other pattern, there were few dominant immunophenotypical variants that remained present throughout the whole culture period. The quantitative relationships between early and late passages are presented in more detail in Figure S1. Furthermore, it should be noted that the evolution of some particular phenotypes was clearly donor-dependent. Donor 1 exhibited particularly significant discrepancies, mostly in Panel 2, but also in Panels 4 and 5 (Figure S2).

**Figure 5:**
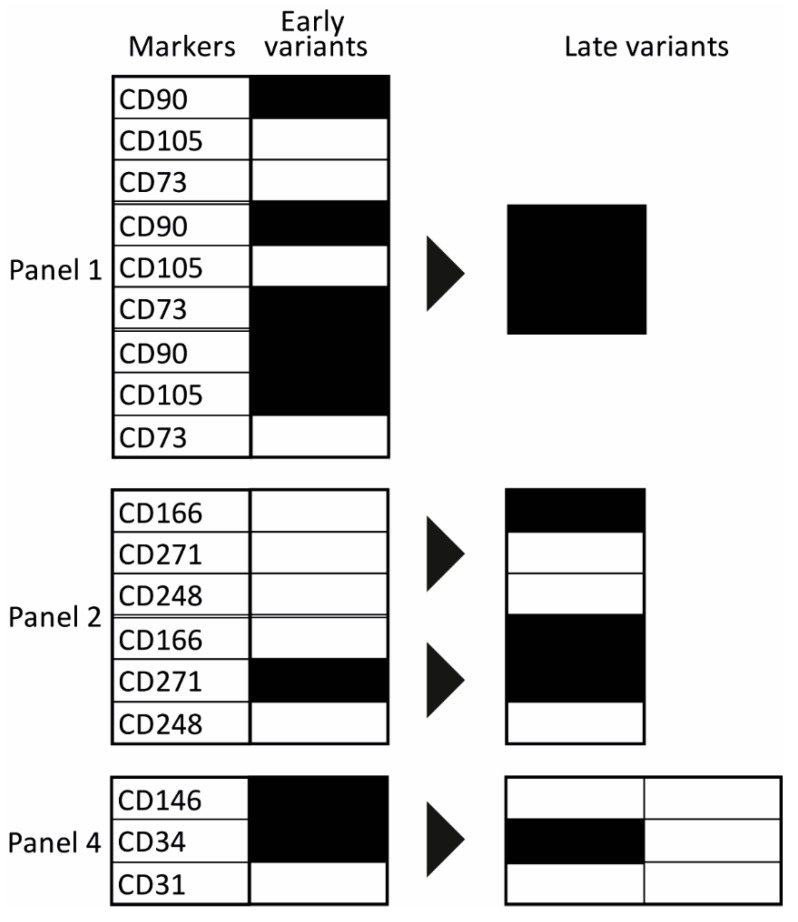
Evolution of predominant phenotypical variants. Panels: 1, mesenchymal stem cell (MSC) markers; 2, wound healing markers; 4, Adipose-derived stromal/stem cell (ASC) markers. A black field indicates that the marker was expressed, a white field indicates that the marker was not expressed. The black arrow reflects the evolution trend from the early stage to later culturing.

In order to obtain a better understanding of the evolutionary processes in the cultures, we looked into what shifts in the marker expression occurred as a result of continued culturing (Figure 5). Regarding Panel 1, a convergence from single- or double-expressed markers to a triple co-expression took place. In Panel 2, the phenotypes switched through the acquisition of single CD166, and in Panel 4, a divergence occurred where the CD146+CD34+ phenotype shed only the CD146 or both the CD146 and CD34.

## 3. Discussion

ASCs hold great promise for emerging cell-based therapies and tissue engineering. Previous research has demonstrated that ASC cultures are actually a mixture of diverse phenotypes, presumably with different functionalities, and with a transcriptional pattern which evolves along with the expansion of the population [39,46]. Moreover, donor variation and fat tissue origin are additional contributing factors that have a major influence on the prevalence of particular immunophenotypes [18,36,47]. It can be speculated that these factors may be the source of variation between studies and the reason for suboptimal outcomes of some pre-clinical and clinical trials [36,48,49,50,51,52] and that it is plausible that by establishing a reliable association between some predictive biomarkers, such as surface membrane immunophenotype and clinical efficacy, ASC potential could be exploited to its fullest in the future. To date, the evolution of complex co-expressed immunophenotypic repertoires within ASC cultures has not been explored in a systematic fashion. Thus, as a first step, we have in the current study invoked 15 well-defined surface epitopes and followed their temporal changes in triple co-expression pattern using fat samples from five independent donors.

Not surprisingly, our investigation of single-marker expression corroborated well-established evidence regarding the uniform expression of hallmark antigens CD73, CD90, CD105, CD166, and CD29, and absence of CD31 in stages following P0 [17,18,46,53,54,55,56]. Nevertheless, some substantial discrepancies in previous data were uncovered with several markers as well. For instance, while the CD34 has initially been claimed not expressed [13] and some authors later were able to detect it in the early stages after primary isolation [46,57], our experiments revealed a robust expression between 30% and 90% (Figure 2). By the same token, in the light of previous reports indicating lack of expression of Stro-1 [20] which in retrospect may be attributed to antibody sources and detection methods [58], we could consistently identify this marker—albeit at a wide span of levels—ranging from around 3% to more than 50%, supporting the findings of Ning et al. and Zuk et al. [59,60]. This may be of biological importance due to Stro-1 being involved in clonogenicity, homing, and angiogenesis [7]. Less dramatic, though still considerable differences in expression levels were noticed for other surface markers, including CD36, CD146, CD200, CD201, and CD271 [18,34,61,62,63]. When using quantitative immunofluorescence techniques, such as multicolour flow cytometry, it is difficult to achieve absolute accuracy, unless highly prevalent and expressed epitopes are searched for. Additionally, results may vary depending on experimental set-up and instrumentation; proper cross-bleed compensation is also critical. Inconsistencies among data from independent laboratories, which can, for the most part, be attributed to donor- and/or tissue-source-related idiosyncrasies, can further be burdened by these experimental artefacts. It is therefore imperative that the subjects and procedures involved in tissue processing and marker analysis be adequately described and standardized as much as possible. Having previously been faced with the pitfalls of the multichromatic fluorescence cell sorting [12], it is our assumption that the discrepancies reported herewith are mostly attributable to the biological nature of our samples.

Despite the fact that our study enabled direct exploration of only triple marker combinations, based on the temporal patterns of individual markers as highlighted above, it is possible to infer that after the initial few passages, all cells came to express the common phenotype CD73+CD90+CD105+CD166+CD29+CD201+. The CD73+CD90+CD105+ triple positivity denotes stem/progenitor cells [13], and it is interesting that these cells became enriched by in vitro growth. Similarly, it is intriguing that a subset of these cells exhibiting the CD31−CD34+CD146− profile, previously found to identify a stem cell lineage [25,64,65], also grew proportionally to the culture period. In addition, possibly important ramifications may reveal that some antigens that have been ambiguously associated with stemness, including CD200, CD248, and CD271 [66,67], were reinforced during the culture, while all subsets featuring CD146, which has previously been recognized as a surrogate for stemness [40,68,69,70,71] were sequestered. Concerning interpersonal variability, the spread of the data roughly follows previously reported trends [49,72], except for two specific subsets, the CD166+CD271−CD248+ and CD166+CD271+CD248+, which were identified only in a single donor. It seems reasonable to assume that similar variations underlie, at least in part, the unique properties observed with some donors [36,52,73,74], but implications for providing a carrier with a selective advantage through a specific biological mechanism remain to be elucidated. In the light of a clinical advantage, researchers are beginning to look into the biological mechanism of specific co-expressed surface marker profiles [75,76,77]. It was found that CD34/CD90 double-positive cells was present after 30-days of expansion and that they may play a vital role in tissue reconstruction. Additionally, we have earlier found the subpopulation CD73+CD90+CD105+CD34-CD146+CD271− to have a positive effect on endothelial cells [12]. Consequently, it is worth continuing investigations of the biological effects of these interesting subsets to gain a more comprehensive understanding of the mechanism of action of ASCs.

Based on our data, it is obvious that even after a major homogenization of the immunophenotypical repertoire taking place at P1, stabilized discrete lineages remain within the culture population that can be discriminated, at least in our case, by the expression of CD34, CD200, CD248, and CD271. It appears worthwhile that the highly defined subpopulations within ASC cultures are to be studied using at least a set of markers as outlined in this study, since such an investigation might reveal hitherto unknown relationships between the immunophenotypical profiles and functionality. This kind of information may be greatly beneficial for improving the efficacy of future ASC therapeutic applications.

## 4. Materials and Methods

### 4.1. ASC Isolation and Expansion

Adipose tissue samples used in this study were derived from five healthy donors who underwent cosmetic liposuction surgery at Aarhus University Hospital, Aarhus, Denmark or Aleris-Hamlet Private Hospital, Aalborg, Denmark. Written informed consent was obtained from each donor prior to donation, and our study was approved by the regional committee on biomedical research ethics on Northern Jutland (Project No. N-20160025, 17 April 2016). Tissue was collected according to Danish legislation on anonymized tissue (Komitélov §14), and the collection complied with the principles defined by the Declaration of Helsinki. ASCs were isolated as described previously in our laboratory [78]. In brief, after being washed four times with sterile phosphate-buffered saline (PBS; Gibco, Taastrup, Denmark), adipose tissue was digested with 0.6 U/mL collagenase NB 4 standard grade (Nordmark Biochemicals, Uetersen, Germany) in Hanks’ Balanced Salt Solution (Gibco, Taastrup, Denmark) for 1 h at 37 °C under continuous agitation. The obtained dissociated tissue was filtered through a 100 μm filter (Millipore, Omaha, NE, USA) followed by low-speed centrifugation at 400× *g* for 10 min. After removing the supernatant, the cell pellet was resuspended in growth media that was alpha-Minimum Essential Medium with GlutaMAX supplemented with 10% fetal calf serum (FCS) and 1% antibiotics (all from Gibco, Taastrup, Denmark), filtered through a 60 μm filter (Millipore Omaha, NE, USA), and, finally, centrifuged at 400 g for 10 min.

The acquired cell pellet was resuspended in the growth medium, and cell yield was determined by the Nucleocounter NC-200 cell counter (Chemometec, Allerod, Denmark). Consequently, the resulting cells were seeded into T175 culture flasks (Greiner Bio-one, Frickenhausen, Germany), and these initial cultures were referred to as passage 0 (P0). At 70%–80% confluency, the cells were detached using TryPLE (Gibco, Taastrup, Denmark), and all the following cultures (P1–P8) were initiated at a density of 5000 cells/cm^2^ [30]. During culture, the medium was changed every 2–3 days.

### 4.2. Multichromatic Flow Cytometry

Initially, ASCs from three donors were analyzed at P1, 2, 4, 6, and 8 in regard to 15 selected surface epitopes. To further examine the range of inter-donor variability, two additional donors were included in passages P1, 4, and 8. The 15 directly-labeled antibodies used to detect the surface epitopes were assorted in five panels of triple combinations based on functionality (Table 1) and used simultaneously with Fixable Viability Stain 570 (FVS570) (BD Biosciences, Lyngby, Denmark) to exclude the dead cells from analysis. For technical details about antibodies, see Table A1. For staining, cell suspensions were first filtered through a cell strainer (70 μm; BD Falcon; BD Bioscience, Erembodegem, Belgium) and subsequently dispensed at 2 × 10^5^ cells per reaction tube. Cells were initially incubated with the viability reagent for 15 min at room temperature, after which a mixture of antibodies optimally diluted in PBS supplemented with 2% FCS and 0.1% sodium azide (Merck Schuchardt, Hohenbrunn, Germany) was added. In Panel 5, where two or more BD Horizon Brilliant dyes were used, BD Horizon Brilliant Stain Buffer (BD Bioscience, Erembodegem, Belgium) was used as a diluent. After staining for 30 min at 4 ℃ in the dark, the samples were washed twice with 50% Accumax (Sigma-Aldrich, Copenhagen, Denmark) in PBS to prevent cell aggregation. For each panel at each passage, two or three independent staining experiments were conducted.

**Figure A1:**
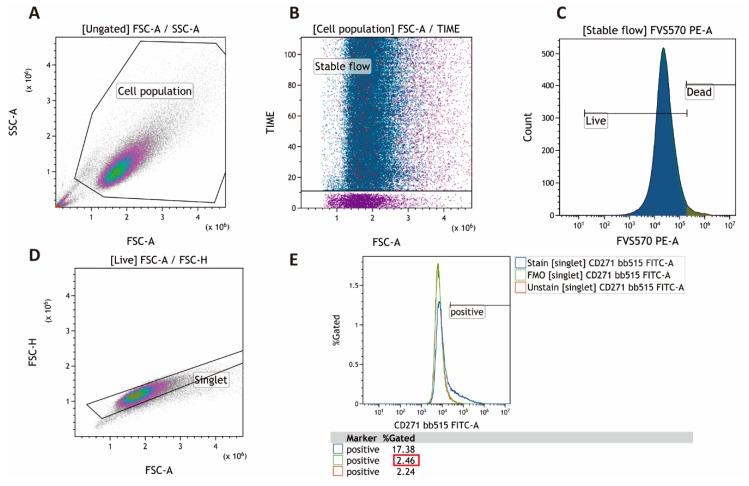
Gating strategy for flow cytometry. (**A**) Noise was removed to collect the cell population using forward scatter area (FSC-A) versus side scatter area (SSC-A). (**B**) To ensure a stable flow during data aquisition, the FSC-A versus time plot was controlled. (**C**) To exclude dead cells, viability dye (FVS570) was used. (**D**) The doublets were discriminated via FSC-A versus forward scatter height (FSC-H). (**E**) Representative of the overlay histogram of the unstained, fluorescence minus one (FMO) and stained group. Red box: the threshold was set at the 2.5% of FMO.

For surface epitope analysis, a CytoFLEX (Beckman coulter, Copenhagen, Denmark) flow cytometer was employed. Prior to analyzing the samples, compensation values were established with the aid of the BD CompBeads Plus Set Anti-mouse Ig, κ and Anti-rat Ig, κ (BD Biosciences, Erembodegem, Belgium) and invoking a compensation matrix based on the dot-plots delineating the combination of channels used in the particular experiment. The data were visualized and analyzed in the Kaluza 2.1 software package (Beckman Coulter, Indianapolis, IN, USA). To validate data analysis, four gates were adopted: one to remove debris (forward scatter area vs. side scatter area); one to ensure cell flow and flow cytometer stability (forward scatter area vs. time); one to discriminate doublets (forward scatter area versus forward scatter height); and one to eliminate dead cell (viability dye intensity histogram). Fluorescence minus one (FMO) controls were used to define the cutoff limit for background values, which was generically set at 97.5th percentile. The gating strategy can be seen in Figure A1.

### 4.3. Statistical Analysis

The data entailing 2–3 replicates for each passage derived from each of the three donors were presented as mean + standard deviation (SD). The General Linear Model (GLM) procedure with repeated measures followed by post-hoc tests of the IBM SPSS Statistics v.26 software package (IBM, Armonk, NY, USA) was utilized to assess the differences between the passages and donors. The level of significance was set at 0.05.

## Figures and Tables

**Table 1:** Panel design for flow cytometry.

Panels					Cytometer Setup		Fluorochrome
1. MSC Markers	2. Wound Healing	3. Immune Regulation	4. ASC Marker	5. Differentiation Capacity	Laser	Emission Channel	
				CD201	405 nm	450/45BP	BV421
CD105						525/40BP	BV510
	CD166			CD36		610/20BP	BV605
		CD29				660/20BP	BV650
			CD146		561 nm	610/20BP	PE-CF594
FVS570	FVS570	FVS570	FVS570	FVS570		585/42BP	Viability dye
	CD271	CD200	CD34			780/60BP	PE-Cy7
	CD248			Stro-1	638 nm	660/20BP	AF647
		CD274				712/25BP	APC-R700
			CD31			780/60BP	APC-Cy7
CD73					488 nm	525/40BP	FITC
CD90						690/50BP	PerCP-Cy5.5

ASC; adipose-derived stromal/stem cells, MSC; mesenchymal stem cell,, BP; band pass, FVS570; fixable viability stain 570, AF647; Alexa Fluor 647.

## Supplementary Materials

Supplementary Materials can be found at https://www.mdpi.com/1422-0067/21/4/1408/s1.

## Abbreviations

ASC	Adipose-derived stromal/stem cells
BP	Band pass
CD	Cluster of differentiation
FCS	Fetal calf serum
FMO	Fluorescence minus one
FSC-A	Forward scatter area
FSC-H	Forward scatter hight
FVS570	Fixable viability stain 570
GLM	General linear model
MSC	Mesenchymal stem cells
P	Passage
SD	Standard deviation
SSC-A	Side scatter area
SVF	Stromal vascular fraction

## Appendix A

**Table A1:** Technical details on antibodies and reagents used for flow cytometry.

**Fluorophore**	**Antigen**	**Host**	**Company**	**Catalog Number**
BV421	CD201	rat	BD Biosciences	743552
BV510	CD105	mouse	BD Biosciences	563264
BV605	CD166	mouse	BD Biosciences	742373
APC-R700	CD274	mouse	BD Biosciences	565188
APC-Cy7	CD31	mouse	BD Biosciences	563653
FITC	CD73	mouse	BD Biosciences	561254
BV605	CD36	mouse	BD Biosciences	563518
BV650	CD29	mouse	BD Biosciences	743785
PE-Cy 7	CD200	mouse	BD Biosciences	562125
PE-Cy7	CD271	mouse	BD Biosciences	562122
Alexa Fluor 647	CD248	mouse	BD Biosciences	564994
Alexa Fluor 647	Stro-1	mouse	R & D system	FAB1038R
Percp-Cy5.5	CD90	mouse	BD Biosciences	561557
PE-CF594	CD146	mouse	BD Biosciences	564327
PE-Cy7	CD34	mouse	BD Biosciences	560710
**Product Name**		**Company**		**Catalog Number**
BD Horizon™ Brilliant Stain Buffer		BD Biosciences		563794
Viability dye, FVS570		BD Biosciences		564995
CompBeads Plus Set Anti-mouse Ig, κ		BD Biosciences		560497
CompBeads Plus Set Anti-rat Ig, κ		BD Biosciences		560499
