# Supplementary material for: Evolution of ASC Immunophenotypical Subsets During Expansion In Vitro
Source: Int J Mol Sci. 2020 Feb 19;21(4):1408. doi: 10.3390/ijms21041408 (PMC7073142; doi:10.3390/ijms21041408)
Supplement: Supplementary file 1 [file ijms-21-01408-s001.pdf]

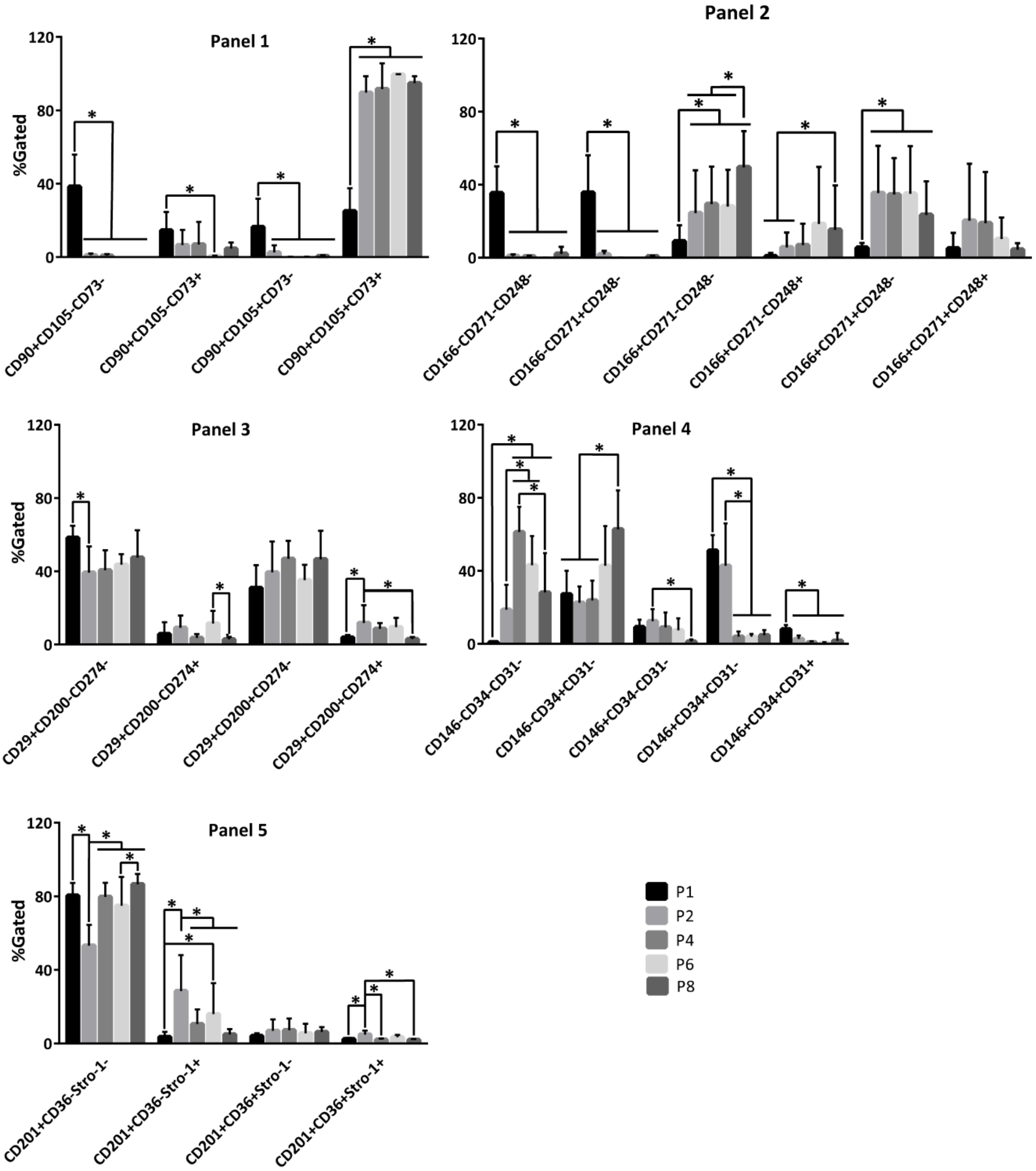

Figure S1. Proportion of adipose-derived stem cells belonging to different subpopulations within each panel of markers. Bars denote mean + standard deviation from 3 independent donors. * $p < 0.05$, P, passage.

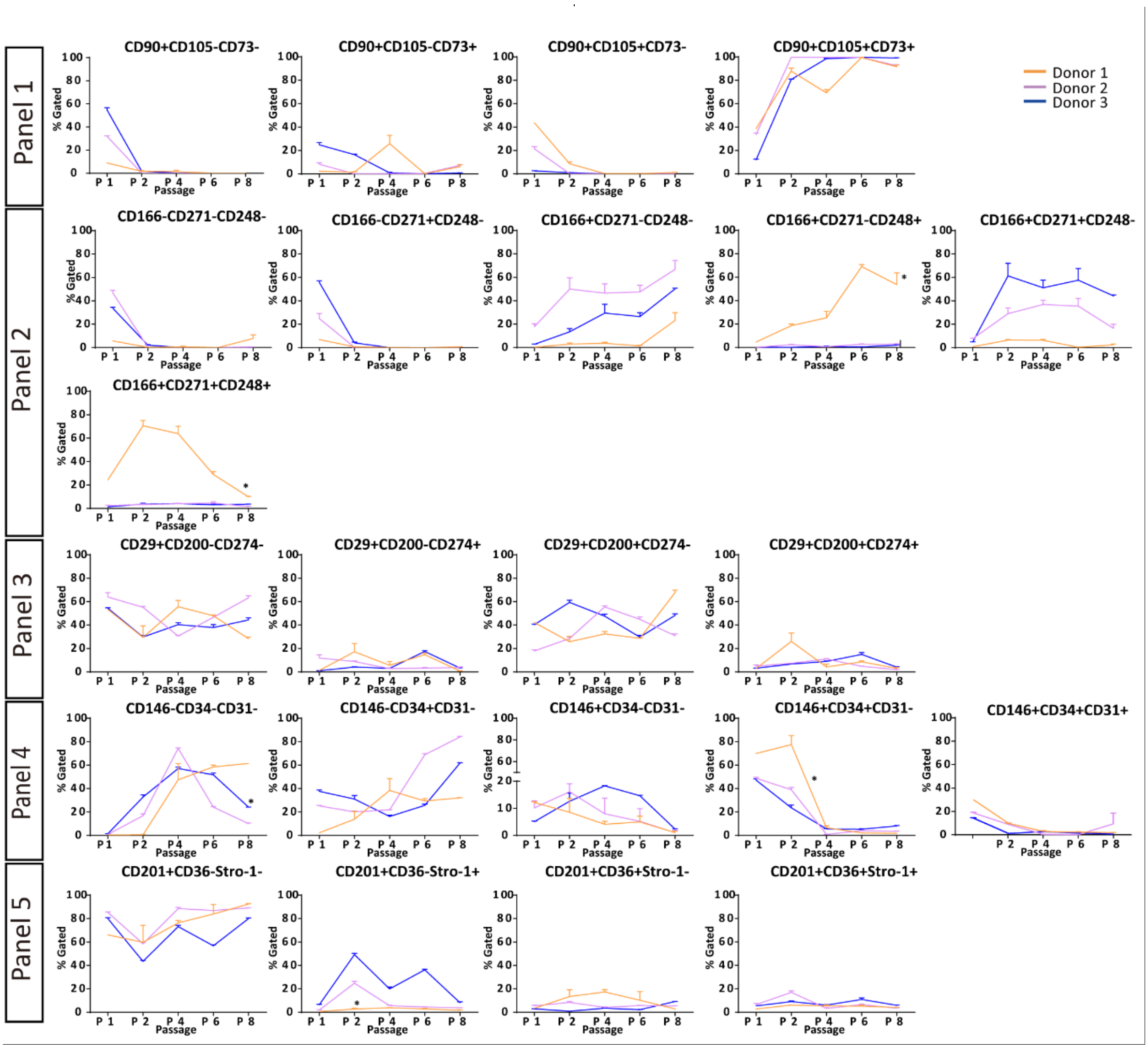

Figure S2. Interdonor variations of dynamic changes to surface marker profiles. Data is presented as average from repeated measurements of each donor (n = 2–3). * denotes statistically significant differences between donor 1 and both donor 2 and 3. $p < 0.05$. P, passage.
